# Natural Variation in *SER1* and *ENA6* Underlie Condition-Specific Growth Defects in *Saccharomyces cerevisiae*

**DOI:** 10.1534/g3.117.300392

**Published:** 2017-11-14

**Authors:** Amy Sirr, Adrian C. Scott, Gareth A. Cromie, Catherine L. Ludlow, Vida Ahyong, Trey S. Morgan, Teresa Gilbert, Aimée M. Dudley

**Affiliations:** * Pacific Northwest Research Institute, Seattle, Washington 98122; † Department of Molecular and Cellular Biology, University of California, Berkeley, California 94720

**Keywords:** yeast, natural variation, QTL mapping, serine metabolism, salt stress

## Abstract

Despite their ubiquitous use in laboratory strains, naturally occurring loss-of-function mutations in genes encoding core metabolic enzymes are relatively rare in wild isolates of *Saccharomyces cerevisiae*. Here, we identify a naturally occurring serine auxotrophy in a sake brewing strain from Japan. Through a cross with a honey wine (white tecc) brewing strain from Ethiopia, we map the minimal medium growth defect to *SER1*, which encodes 3-phosphoserine aminotransferase and is orthologous to the human disease gene, *PSAT1*. To investigate the impact of this polymorphism under conditions of abundant external nutrients, we examine growth in rich medium alone or with additional stresses, including the drugs caffeine and rapamycin and relatively high concentrations of copper, salt, and ethanol. Consistent with studies that found widespread effects of different auxotrophies on RNA expression patterns in rich media, we find that the *SER1* loss-of-function allele dominates the quantitative trait locus (QTL) landscape under many of these conditions, with a notable exacerbation of the effect in the presence of rapamycin and caffeine. We also identify a major-effect QTL associated with growth on salt that maps to the gene encoding the sodium exporter, *ENA6*. We demonstrate that the salt phenotype is largely driven by variation in the *ENA6* promoter, which harbors a deletion that removes binding sites for the Mig1 and Nrg1 transcriptional repressors. Thus, our results identify natural variation associated with both coding and regulatory regions of the genome that underlie strong growth phenotypes.

The biological consequences of perturbing a metabolic pathway can be severe. In humans, polymorphisms in genes that encode core metabolic enzymes can cause rare, monogenic diseases known collectively as inborn errors of metabolism. These disorders generally result from loss-of-function mutations within the coding region of metabolic genes that lead to either a toxic buildup of precursor metabolites or deficiencies in the reaction end products (Lanpher *et al.* 2006; El-Hattab 2015; Vernon 2015). While often fatal if untreated, in many cases timely diagnosis followed by appropriate dietary restrictions or supplementation can greatly reduce or even eliminate symptoms in these patients (Vernon 2015). Serine deficiency disorders provide one such example. Disruption of any one of the three enzymes (3-phosphoglycerate dehydrogenase, phosphoserine aminotransferase, and phosphoserine phosphatase) in the phosphorylated serine biosynthesis pathway causes severe neurodevelopmental disorders that present with congenital microcephaly, intractable seizures, and psychomotor retardation (Acuna-Hidalgo *et al.* 2014; El-Hattab *et al.* 2016). However, case studies have shown that morbidity and mortality may be reduced and even eliminated with serine supplementation (de Koning *et al.* 2002, 2004; Hart *et al.* 2007; Brassier *et al.* 2016). In one such case, prenatal diagnosis of 3- phosphoglycerate dehydrogenase deficiency followed by L-serine supplementation to the mother early in pregnancy (week 27) and to the patient from birth onward completely prevented the onset of neurological symptoms (de Koning *et al.* 2004).

In the model organism *Saccharomyces cerevisiae*, gene deletions or loss-of-function mutations in amino acid or nucleobase biosynthetic enzymes are widely used as genetic markers and tools for genetic engineering. Accordingly, standard growth conditions include abundant supplies of the nutrients required to support the robust growth of both prototrophic and auxotrophic strains (Rose *et al.* 1990). Whether yeast cells synthesize a metabolite or import it from the surrounding environment depends on many factors, including the interplay between the nutrient sensors monitoring the metabolic status of the cell and signaling cascades that regulate metabolic pathways (Conrad *et al.* 2014; Palm and Thompson 2017). When a biosynthetic pathway is disrupted and no secondary route exists, cells depend entirely on exogenous supplies of the missing metabolite. However, nutritional supplementation does not always fully compensate for impaired biosynthesis (Pronk 2002), and auxotrophies have been shown to have widespread effects on the transcriptional profile of a cell, even when grown in rich conditions (Ronald and Akey 2007; Alam *et al.* 2016).

Because naturally occurring auxotrophies in *S. cerevisiae* are thought to be rare, the influence of auxotrophies on phenotypic traits has largely been investigated in laboratory strains. However, a number of groups have begun to assemble and characterize collections of natural isolates, thereby sampling the natural variation within the species across a wide range of habitats (Fay and Benavides 2005; Liti *et al.* 2009; Schacherer *et al.* 2009; Goddard *et al.* 2010; Cromie *et al.* 2013; Strope *et al.* 2015; Ludlow *et al.* 2016). One powerful statistical method for studying the functional consequences of natural variation is quantitative trait locus (QTL) mapping (Lander and Botstein 1989; Kruglyak and Lander 1995). This large-scale mapping approach looks for statistical associations between genotype and phenotype information from relatively large numbers of recombinant progeny, with the goal of identifying common regions of the genome associated with a trait.

Here, we describe a cross between two yeast strains isolated from alcoholic beverages, but with genetically and geographically distinct lineages (Fay and Benavides 2005; Liti *et al.* 2006; Cromie *et al.* 2013): an auxotrophic strain isolated from Japanese sake and a prototrophic strain isolated from Ethiopian honey wine. Ethiopian honey wine (white tecc) is traditionally made in small batches and fermented by spontaneous cultures with batch-to-batch variation in microbial content (Bahiru *et al.* 2006). In contrast, the industrialized process of sake brewing relies on inoculations with defined starter cultures of *S. cerevisiae* that have been selected for many traits, including ethanol tolerance, flavor production, and foaming characteristics (Akao *et al.* 2011). While the *S. cerevisiae* strains used in the production of sake are generally prototrophic diploids, chemical and UV mutagenesis methods have been used to isolate auxotrophic haploids for strain construction purposes (Hashimoto *et al.* 2005). Approximately 5% of the progeny of this cross exhibit a range of complex colony morphologies on standard growth conditions (YPD, 2% glucose), and two of the progeny strains from this cross have been used by our laboratory to investigate traits relevant to biofilm formation (Tan *et al.* 2013; Cromie *et al.* 2017a,b).

In this study, we measure the growth of several hundred haploid, recombinant progeny of this cross and use genetic mapping to identify *SER1* as the causative gene underlying the minimal medium growth defect. We also find that *SER1*, which encodes an enzyme in the phosphorylated serine biosynthesis pathway, dominates the QTL landscape even in the presence of abundant external nutrients. We identify a second major-effect QTL associated with growth on salt and map it to the sodium exporter, *ENA6*. We demonstrate that, despite significant variation within the coding region, the growth defect in the presence of salt is largely the result of polymorphisms in the *ENA6* promoter.

## Materials and Methods

### Yeast growth and genetic manipulation

Unless noted, standard media and methods were used for the growth and manipulation of yeast (Rose *et al.* 1990). The *S. cerevisiae* strains used in this study are listed in Table 1. The haploid parental strains of the yeast cross were derived from the UC5 (Fay and Benavides 2005) sake brewing strain from Japan (hereafter the “sake” genetic background) and the DBVPG1853 (Liti *et al.* 2006) white tecc brewing strain from Ethiopia (hereafter the “tecc” genetic background). Following mating of the sake (YO486) and tecc (YO502) parental strains and sporulation of the heterozygous diploid (Tong *et al.* 2001), a total of 552 recombinant progeny were isolated by manual tetrad dissection on YPD agar plates.

**Table 1 S.** *cerevisiae* strains used in this study

Strain	Background	Genotype	Description	Source
UC5 (YO300)	Sake	MAT**a**	Original isolate	Fay and Benavides (2005)
DBVPG1853 (YO8)	Tecc	MAT**a**/MATα	Original isolate	Liti *et al.* (2006)
YO607	Sake	MAT**a** *ho*Δ*0*::*hphMX6*	Sake with *HO* deleted	This study
YO486	Sake	MAT**a** *ho*Δ*0*::*hphMX6 SPS2:EGFP:kanMX4*	Sake parent of cross	This study
YO601	Tecc	MAT**a**/MATα *ho*Δ*0*::*hphMX6/HO*	Tecc with *HO* deleted	This study
YO560	Tecc	MATα *ho*Δ*0*::*hphMX6*	Tecc haploid isolate with *HO* deleted	This study
YO502	Tecc	MATα *ho*Δ*0*::*hphMX6 SPS2:EGFP:natMX4*	Tecc parent of cross	This study
YPG337-YPG888	Sake × tecc	*ho*Δ*0*::*hphMX6*, (*SPS2:EGFP:kanMX4* or *SPS2:EGFP:natMX6*)	Sake × tecc cross progeny	This study
YO2331, YO2530, YO2531	Sake	MAT**a** *SER1-S:kanMX4*	Sake with *SER1* drug marker control	This study
YO2319, YO2320	Tecc	MATα *ho*Δ*0*::*hphMX6 SER1-T:kanMX4*	Tecc with *SER1* drug marker control	This study
YO2430, YO2431	Sake	MAT**a** *ser1*Δ*0*::*natMX6*	Sake with *ser1* deletion	This study
YO2571, YO2572	Tecc	MATα *ho*Δ*0*::*hphMX6 ser1*Δ*0*::*natMX6*	Tecc with *ser1* deletion	This study
YO2328, YO2329	Sake	MAT**a** *SER1-T:kanMX4*	Sake with tecc *SER1* allele swap	This study
YO2316, YO2317	Tecc	MATα *ho*Δ*0*::*hphMX6 SER1-S:kanMX4*	Tecc with sake *SER1* allele swap	This study
YO2402-YO2404	Sake	MAT**a** *ENA6:natMX6*	Sake with *ENA6* drug marker control	This study
YO2432, YO2433	Tecc	MATα *ho*Δ*0*::*hphMX6 ENA6:natMX6*	Tecc with *ENA6* drug marker control	This study
YO2463, YO2464	Sake	MAT**a** *ena6*Δ*0*::*natMX6*	Sake with *ena6* deletion	This study
YO2465, YO2466	Tecc	MATα *ho*Δ*0*::*hphMX6 ena6*Δ*0*::*natMX6*	Tecc with *ena6* deletion	This study
YO2524	Sake	MAT**a** *ENA6-pS-T:natMX6*	Sake with *ENA6-pS-T* allele swap	This study
YO2525, YO2526	Sake	MAT**a** *ENA6-pS1-T:natMX6*	Sake with *ENA6-pS1-T* allele swap	This study
YO2527, YO2528	Tecc	MATα *ho*Δ*0*::*hphMX6 ENA6-pT-S:natMX6*	Tecc with *ENA6-pT-S* allele swap	This study
YO2719, YO2722	Tecc	MATα *ho*Δ*0*::*hphMX6 ENA6-pS-T:natMX6*	Tecc with *ENA6-pS-T* promoter swap	This study
YO2716, YO2717	Tecc	MATα *ho*Δ*0*::*hphMX6 ENA6-pS1-T:natMX6*	Tecc with *ENA6-pS1-T* promoter swap	This study

### Yeast strain construction

Haploid strains for the sake and tecc cross were generated as follows. First, UC5 and DBVPG1853 were transformed with an *ho*Δ*0*::*hphMX6* cassette (Sirr *et al.* 2015) to produce a sake heterothallic MAT**a** haploid (YO607) and a tecc MAT**a**/α diploid that was hemizygous at the *HO* locus (YO601). Next, YO601 was sporulated and a MATα heterothallic haploid resistant to hygromycin (YO560) was selected from hand-dissected tetrads. The final strains used in the cross (YO486 and YO502) were generated by transforming YO607 with *SPS2:EGFP:kanMX4* and YO560 with *SPS2:EGFP:natMX4* as previously described (Sirr *et al.* 2015). Since the original UC5 isolate (YO300) harbored a nonfunctional *HO* gene (C. L. Ludlow and A. M. Dudley, unpublished results), it was subsequently used as the sake genetic background strain for some strain construction and growth assays.

*SER1* was deleted from the sake and tecc genetic backgrounds as follows. First, the drug marker of the *ser1*Δ*0*::*kanMX* allele in the yeast deletion collection (Winzeler *et al.* 1999) was switched from *kanMX* to *natMX* by transformation as previously described (Goldstein and McCusker 1999). The resulting *ser1*Δ*0*::*natMX* cassette was then PCR amplified using primers SER1_del_F and SER1_del_R (Supplemental Material, Table S1) and transformed into YO300 (sake) and YO560 (tecc).

To generate *SER1* allele-replacement and control strains, integrating plasmids harboring alleles of *SER1* (Table S2) were constructed using the New England BioLabs HiFi DNA Assembly Kit. Primers (Table S3) were designed using the NEBuilder Assembly Tool (http://nebuilder.neb.com/). Plasmid pAS11-SER1-S contains the sake *SER1* allele (*SER1-S*) which consists of the sake *SER1* open reading frame, 95 bp upstream of the start codon, 350 bp downstream of the stop codon, and the *kanMX4* drug marker followed by 240 bp of homology to a region downstream of *SER1* (to direct integration). The *SER1-S* allele [PCR amplified from YO300 using primers pUC19_SER1_F and SER1_R (Table S3)] and the *kanMX4* cassette gene fragments [either synthesized by GENEWIZ or PCR amplified using primers TEF_promoter_F and TEF_terminator_R (Table S3)] were cloned into a *Bam*HI/*Pst*I-digested pUC19 vector backbone. Plasmid pAS12-SER1-T was assembled in the same way, except using the tecc allele of *SER1* [*SER1-T*, PCR amplified from YO560 using primers pUC19_SER1_F and SER1_R (Table S3)]. *SER1-S:kanMX4* was liberated from pAS11-SER1-S by *Sna*BI/*Sap*I double digestion and transformed into both the sake genetic background (to generate *kanMX*-tagged control strains) and into the tecc genetic background (to generate allele-replacement strains). *SER1-T:kanMX4* was liberated from pAS12-SER1-T by *Sna*BI/*Sap*I double digestion and transformed into both the tecc genetic background (to generate *kanMX*-tagged control strains) and into the sake genetic background (to generate allele-replacement strains) (Table 1). Transformants harboring the correct integration of the *SER1:kanMX4* cassette were identified by PCR and Sanger sequencing (GENEWIZ).

Sake and tecc *ENA6:natMX6* control strains (Table 1) were constructed by transforming *natMX6* cassettes PCR amplified with primers ENA_300_tag_F or ENA_560_tag_F and ENA_tag_R (Table S1) into YO300 and YO560, respectively. The *ENA6* deletion strains were constructed using *kanMX4* cassettes PCR amplified with primers ENA_del_F and ENA_del_R (Table S1) and transformed into YO300 and YO560.

For the *ENA6* allele swaps, the tecc *ENA6* allele (*ENA6-T*), including 234 bp upstream of the start codon, the complete open reading frame, and 349 bp downstream of the stop codon was PCR amplified from the *kanMX*-tagged control strain YO2432 using primers ENA_swap_F and ENA_swap_R (Table S1) and transformed into the sake *ena6*Δ strain YO2463. Integration of the tecc *ENA6:natMX6* cassette into the sake background was confirmed by PCR and Sanger sequencing. Different points of integration resulted in two allele variants in the sake genetic background. In isolate YO2524, the *ENA6* coding region is identical to tecc *ENA6*, but the promoter region is identical to the sake genetic background (Table S4). We refer to this allele as *ENA6-pS-T*. The *ENA6* coding regions of isolates YO2525 and YO2526 are also identical to the tecc *ENA6*, but these strains have tecc SNPs present in the first ∼234 bp of the promoter (Table S4), resulting in a sake/tecc hybrid promoter. We refer to this allele as *ENA6-pS1-T*.

The reciprocal swap of the sake *ENA6* allele into the tecc background was constructed in the same fashion. The sake *ENA6* allele (*ENA6-S*) was PCR amplified from the *kanMX*-tagged control strain YO2402 using primers ENA_swap_F and ENA_swap_R (Table S1) and transformed into the tecc *ena6*Δ strain YO2465. Proper integration of the sake *ENA6:natMX6* cassette into the tecc background was confirmed by PCR and Sanger sequencing. We refer to this allele as *ENA6-pT-S*.

For allele replacements of the *ENA6* promoter region, a longer segment containing the sake *ENA6* upstream repressor sequence (URS) (Serrano *et al.* 2002) and sake/tecc hybrid URS region was integrated into the tecc genetic background as follows. Primers ENA_swap_F2 and ENA_swap_R (Table S1) were used to PCR amplify the entire *natMX6*-tagged *ENA6-pS-T* allele from YO2524. This allele, containing 610 bp of the sake promoter region and the tecc *ENA6* open reading frame, was then transformed into the tecc *ena6*Δ strain YO2465. Additionally, the same primers were used to PCR amplify the *ENA6-pS1-T* allele from YO2525, which produced a sake/tecc hybrid promoter. This allele consists of the tecc promoter sequence for the first ∼234 bp upstream of the *ENA6* start, followed by 376 bp of the sake promoter. These cassettes were integrated into the tecc *ena6*Δ strain YO2465. Tecc *ENA6* strains with the extended sake promoter allele (*ENA6-pS-T)* and tecc *ENA6* strains with the extended sake/tecc hybrid promoter allele (*ENA6-pS1-T)* were PCR and sequence confirmed (Table 1 and Table S4).

### Yeast growth assays

Cross progeny strain growth was assayed on different media (Table 2) as follows. Strains arrayed in a 96-well format were grown overnight at 30° in liquid YPD. For all conditions except SD, strains were replica pinned onto solid media in 250-mm square bioassay trays (Teknova, Hollister, CA) at a density of six well plates per bioassay tray using a QP Expression robot (Molecular Devices, Sunnyvale, CA). For growth on SD, strains were manually pinned onto 127 × 85 mm agar plates. Strains were grown at 30° and imaged once daily for 7 d.

**Table 2:** Solid media used in this study

Condition	Composition
Rich medium	YPD (2% glucose)
Copper	YPD (2% glucose) + 2.5 mM CuSO_4_
Ethanol	YPD (2% glucose) + 10% EtOH
Sodium	YPD (2% glucose) + 0.5 M NaCl
Rapamycin	YPD (2% glucose) + 0.1 µg/ml rapamycin
Caffeine	YPD (2% glucose) + 10 mM caffeine
YPD + Kan*^a^*	YPD (2% glucose) + 200 µg/ml G418
YPD + Nat*^a^*	YPD (2% glucose) + 100 µg/ml nourseothricin
YPD + Hyg*^a^*	YPD (2% glucose) + 500 µg/ml hygromycin
Minimal medium	SD (2% glucose)
SD + serine	SD (2% glucose) + 1 mM serine

a Drug plates used for verifying strain construction.

Growth of strains harboring deletions or allele replacements of the *SER1* and *ENA6* candidate genes was assayed by arraying YPD cultures grown overnight at 30° into a checkerboard pattern, such that the pinning density was 48 evenly spaced strains per 127 × 85 mm agar plate. Plates were grown at 30° for 3 d and photographed daily. Growth was measured as patch area extracted from the images using a custom script (File S2) for ImageJ (Schneider *et al.* 2012). Briefly, for each image, the plate corners were manually identified and patch locations were interpolated from the plate layout. Then, the plate corners and patch edges were enhanced using a variance filter. The edges were converted to a binary mask and filled, and the area of each filled region [in square pixels (px^2^)] was used as the patch area. Patch areas were converted from px^2^ to mm^2^, and the area in mm^2^ for each day was used for QTL mapping.

### Genotyping

Parental strains of the cross were whole genome sequenced using 2 × 36 bp (tecc) and 2 × 80 bp (sake) paired-end reads on an Illumina GA II. Genomic DNA was isolated using a YeaStar Genomic DNA Extraction Kit (Zymo Research). DNA sequencing libraries were prepared using the Paired-End Sequencing Kit (Illumina) following the manufacturer’s instructions. Reads were aligned to the S288c reference genome (R64-1-1) using BWA (v5.8) (Li and Durbin 2009), allowing six mismatches and using quality trimming (threshold of Phred = 20). SAMtools (v0.1.18) (Li *et al.* 2009) was then used to generate a pileup file for each parental strain, using the -C 50 and -q 20 parameters. At each position in the reference genome, the most common base was identified for each strain from the pileup files, with the criterion that the most commonly observed base must be at least five times more frequent than the second most frequent. Insertions and deletions were excluded. Positions that differed between YO486 and YO502 were then identified, allowing construction of a SNP table (Table S5) that defined potential genotyping markers.

Progeny strains were genotyped using RAD-seq (Ludlow *et al.* 2013) and the reads were aligned as above. SAMtools (v0.1.8) (Li *et al.* 2009) was then used to generate a pileup file for each progeny strain. To produce a strain-by-marker genotype table, at each position identified as polymorphic between the two parents in the parental SNP table, the counts of the two parental bases were recorded from each pileup file. This resulted in 5848 unique marker positions. Next, strains with poor coverage were dropped by removing any strains which had reads assigned to <750 marker positions. At each marker position, the remaining strains were assigned the genotype of whichever parent was most frequently represented in the strain’s reads, so long as the most prevalent parent’s reads were at least five times greater than those corresponding to the other parent. Finally, markers were dropped if they had reads in <5% of the strains, or if the ratio of sake calls to tecc calls deviated more than twofold from a 1:1 ratio. After this filtering, there were 457 strains and 537 markers.

### Detecting aneuploidy

The relative chromosome copy numbers were calculated as the proportion of reads in each strain aligned to that chromosome, normalized by the median proportion for that chromosome across all strains (based on the assumption that most strains are euploid). Because half of the progeny strains were disomic for chromosome I, the proportion of reads aligned to chromosome I was normalized to the mean coverage over all strains divided by 1.5. Finally, each strain was normalized such that the median relative chromosome copy number in that strain was equal to one. Strains with a chromosome I coverage value between 1.5 and 2.5 were classed as chromosome I disomes, while strains with a value between 0.5 and 1.5 were classed as euploids/haploids. Strains with values outside the range of 0.5–2.5 were assumed to reflect sequencing errors. Table S6 lists the estimated ploidy of each chromosome in each strain, and strains with a value of one for all chromosomes are considered euploid.

### QTL mapping

QTL were identified using the R/qtl package (Broman *et al.* 2003). Since some of the conditions substantially restricted growth for a subset of the strains, resulting in nonnormal phenotype distributions, a nonparametric model was used. The 552 progeny strains from the cross were filtered by genotyping quality (as described above) and were additionally filtered by the drug resistance from the marker linked to the *SPS2* locus. Specifically, 25 strains which grew larger than 5 mm^2^ after 2 d on both YPD + Kan and YPD + Nat were removed. Also, four strains were removed because they exhibited no growth on YPD after 4 d. Finally, 16 strains were removed because their genotypes were extremely similar (>90% identical) to another strain. As a result, a final set of 412 strains was used for QTL mapping. Table S7 lists the phenotypes and genotypes for all 412 progeny strains as well as the two parents. Significance thresholds for QTL were determined by permutation tests (*n* = 10,000; α = 0.01) in R/qtl. Unless noted, all LOD scores were calculated based on phenotype data acquired on day 4 of growth at 30°.

The non-normal phenotype distributions precluded a stepwise approach based on linear model fitting for uncovering secondary QTL. As an alternative, we repeated the QTL analysis after splitting the population based on the major-effect QTL genotype. For each phenotype, the marker with the highest LOD score was selected and subsets were created for strains with either the sake allele or the tecc allele at that marker. Strains with no allele call were ignored. A single-locus scan was run on each subset, and significance thresholds were calculated by permutation tests for each subset (*n* = 1000, α = 0.01).

### Quantile normalization for interaction tests

Similarities in the QTL mapping results for YPD and three of the YPD-based conditions led us to test whether there was any additional effect of the perturbation beyond the growth defects in YPD. For each of these conditions, we performed two-factor ANOVA using patch area as the response. The two factors were the chr15_643999 allele and the presence or absence of the medium additive (copper, caffeine, or rapamycin). The phenotype distributions on caffeine and rapamycin were bimodal with a distinct floor comprised of strains with no growth. By splitting the population based on the chr15_643999 allele (as in the above secondary QTL analysis) we found that each subset approximated a normal distribution with a floor. We inferred values for strains near this floor by carrying out quantile normalization (Table S8) to a normal distribution with parameters estimated from linear regression against the portion of each subpopulation that was well separated from the floor (growth >5 mm^2^).

### Heritability and genetic variance calculations

We estimated the broad-sense heritability as *H*^2^ = 1−(σ^2^_e_/σ^2^_s_), where σ^2^_s_ is the variance of the population of segregant means, and σ^2^_e_ is the variance of the sampling distribution of these means. We calculated σ^2^_e_ using the random effects ANOVA approach of Bloom *et al.* (2013). First, the pooled within-group (segregant) variance σ^2^_w_ was calculated using the lmer command in R (Bates *et al.* 2015), fitting the random effects model to all segregant individual phenotype measurements where the measurement was >5 mm^2^ and there were at least two such measurements from the segregant. We then calculated σ^2^_e_ as (1/*n* × σ^2^_w_) where *n* is the mean number of phenotype measurements per segregant.

To estimate genetic variance accounted for by the effect of a genetic locus, we normalized the phenotypes of each of the two subpopulations determined by the alleles of that locus to have means of zero. We then calculated the variance of the normalized segregant means σ^2^_ns_ and the genetic variance accounted for as σ^2^_s_−σ^2^_ns_.

### Data availability

All strains and plasmids are available upon request. File S1 contains a description of all supplemental files. Whole genome sequencing data for the sake (YO486) and tecc (YO502) parental strains has been deposited in the Sequence Read Archive under accession numbers PRJEB68822 and PRJEB22489, respectively. RAD-seq data for the progeny has been deposited in the Sequence Read Archive under accession number PRJEB22560. The script used to extract patch areas from images is included as File S2. The R scripts to create the R/qtl cross object and perform analysis, including quantile normalization and heritability calculations, are included with necessary data files as File S3.

## Results

### Coding polymorphism in SER1 underlies the sake auxotrophy

**Figure 1:**
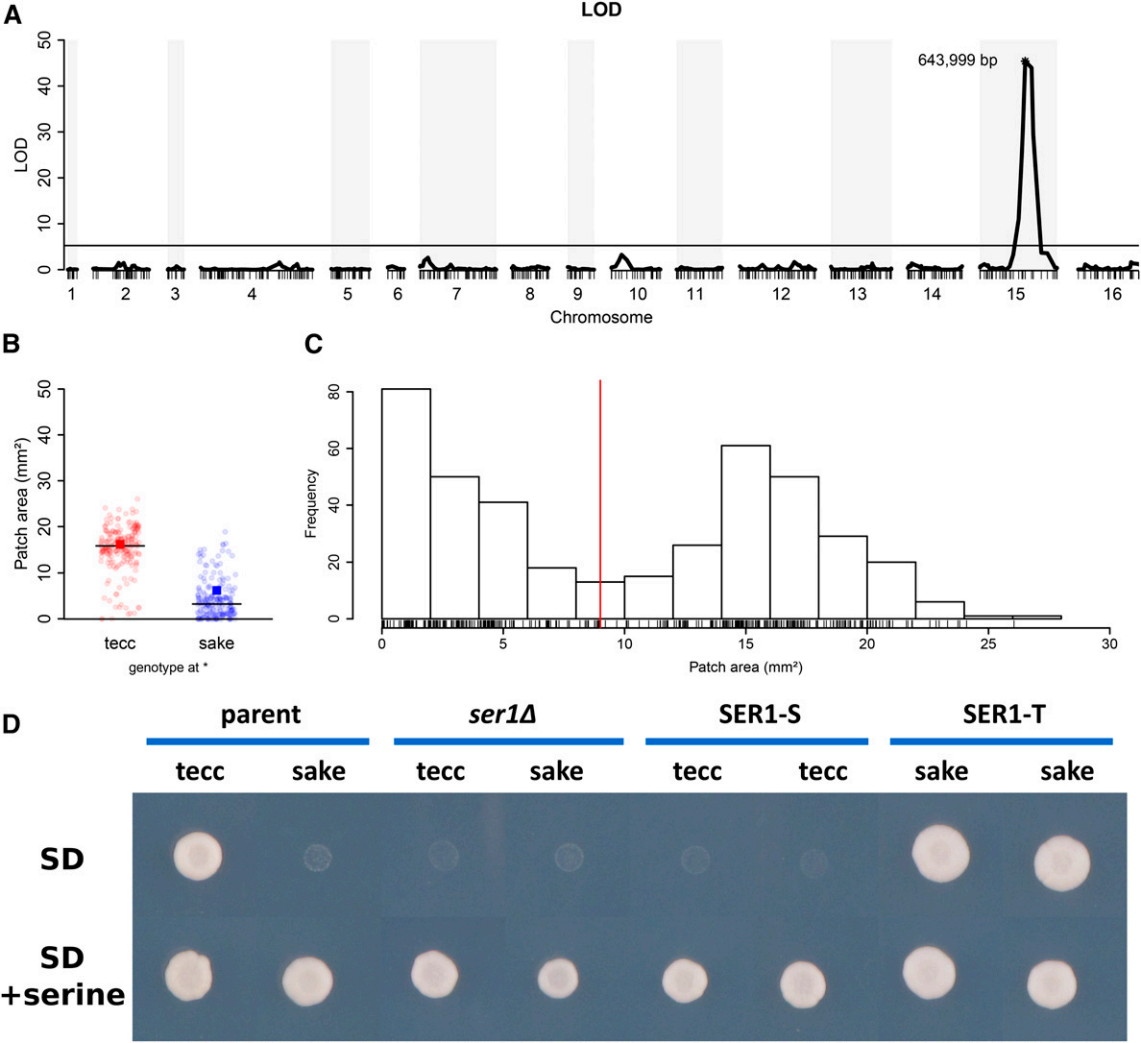
*SER1* is the quantitative trait gene associated with the UC5 auxotrophy. (A) QTL mapping of 412 recombinant progeny grown on minimal medium (SD) identified a single highly significant peak (LOD = 45.4) at the marker at 643,999 bp on chromosome XV. The horizontal line is the significance threshold determined by permutation tests (*n* = 10,000; α = 0.01) in R/qtl. The LOD scores of other statistically significant markers are presented in Table S9. (B) Growth of the progeny strains on SD as measured by patch area and split into subsets based on genotype at 643,999 bp on chromosome XV (tecc allele in red; sake allele in blue). Horizontal lines denote the median value for each group of strains. The growth value of the corresponding parent strain is indicated by a red or blue box. (C) Histogram of progeny strain growth on minimal media, which shows a bimodal pattern. The red line at 9 mm^2^ indicates the approximate minimum between the two growth classes, with 201 strains below that threshold and 211 above. (D) Growth of strains on SD with and without the addition of 1 mM serine, including the parent strains of the cross (tecc and sake), both strains with the *SER1* open reading frame deleted (*ser1*Δ), and two independent isolates of each strain harboring allele replacements of *SER1*. Strains were photographed after 3 d of growth at 30°. Each strain was assayed at least four times across two replicate plates for each condition and a representative example is shown. Additional replicates are shown in Figure S1. Plate images were cropped without resizing or additional image manipulation to create the composite image.

Because naturally occurring auxotrophies are relatively rare in wild isolates of *S. cerevisiae*, we sought to identify the gene(s) underlying the failure of the Japanese UC5 sake strain to grow on minimal medium and the relationship of this trait to other phenotypes by meiotic mapping. We crossed modified versions (*Materials and Methods*) of UC5 (Fay and Benavides 2005) to a genetically distant, prototrophic, Ethiopian white tecc brewing strain DBVPG1853 (Liti *et al.* 2006) and isolated the haploid progeny of 138 three- or four-spore viable tetrads. To determine the gene(s) underlying the sake auxotrophy, we measured growth of the parental strains and 412 progeny on minimal medium (SD) and performed QTL analysis. Our results showed a QTL peak (LOD 45.4) on chromosome XV (Figure 1A and Table S9) that was strongly associated with the minimal medium growth phenotype (Figure 1B). In addition, the distribution of progeny growth on SD (Figure 1C) was consistent with the monogenic segregation of this trait (Hou *et al.* 2016). Examining the functional annotation of the genes in this region suggested a strong candidate gene, *SER1*, which encodes a highly conserved 3-phosphoserine aminotransferase required for the biosynthesis of serine (Ulane and Ogur 1972; Melcher and Entian 1992; Melcher *et al.* 1995). The sake parent of the cross harbors a nonsynonymous coding allele (G78R) (Table S2) that is unique among the strains currently listed in the yeast genome database [SGD project; http://yeastgenome.org/cgi-bin/FUNGI/alignment.pl?locus=YOR184W]. This polymorphism alters an amino acid in a highly conserved region of the protein near the cofactor (pyridoxal phosphate) binding pocket that is essential for enzyme function (Hester *et al.* 1999) and is the only polymorphism within the *SER1* coding region that differs from the amino acid sequence of the reference strain or the tecc strain background.

Consistent with the hypothesis that the sake background harbored a defect in serine biosynthesis, supplementation of the minimal medium with 1 mM serine restored growth of the sake parental strain (Figure 1D and Figure S1). To test whether *SER1* was in fact the causative gene underlying the sake strain auxotrophy, we constructed a series of allele-replacement strains and assayed their growth on minimal medium in the presence and absence of exogenous serine. The results (Figure 1D) show that, in either genetic background, the tecc allele of *SER1* (*SER1-T*) is required for growth on minimal medium and the sake allele (*SER1-S*) confers a serine auxotrophy. Furthermore, the comparable growth defects of *SER1-S* and *ser1*Δ in the absence of serine (Figure 1D) are consistent with the hypothesis that the G78R amino acid substitution confers a loss-of-function phenotype.

### Major-effect QTL common to several conditions

**Figure 2:**
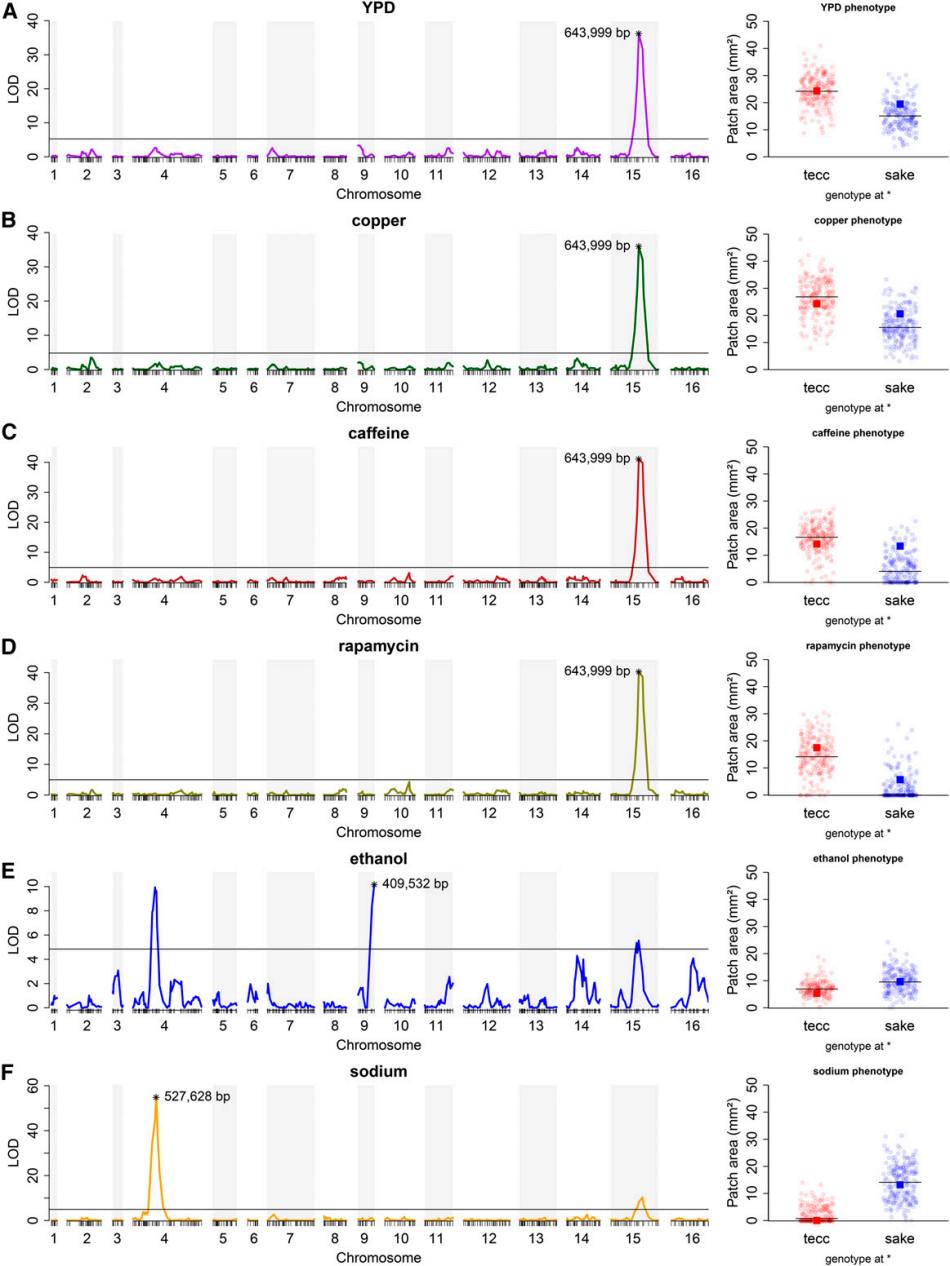
QTL mapping reveals a major QTL peak common to several conditions. QTL mapping of 412 recombinant progeny (left) and the distribution of the growth of the progeny strains split into subsets based on genotype (right) at the marker of the strongest QTL peak (*) for that condition on (A) YPD (LOD = 36.2; marker 643,999 bp on chromosome XV), (B) YPD + copper (LOD = 36.0; marker 643,999 bp on chromosome XV), (C) YPD + caffeine (LOD = 41.0; marker 643,999 bp on chromosome XV), (D) YPD + rapamycin (LOD = 40.2; marker 643,999 bp on chromosome XV), (E) YPD + ethanol (LOD = 10.2; marker 409,532 bp on chromosome IX), and (F) YPD + sodium (LOD = 54.8; marker 527,628 bp on chromosome IV). The horizontal line in QTL plots represents the significance threshold determined by permutation tests (*n* = 10,000; α = 0.01) in R/qtl. The LOD scores of other statistically significant markers are presented in Table S9. Progeny strain growth on each condition was measured by patch area and split into subsets based on genotype (tecc allele in red; sake allele in blue) at the marker with the highest LOD score. Horizontal lines denote the median value for each group of strains. The growth value of the corresponding parent strain is indicated by a red or blue box.

To explore the influence of this naturally occurring polymorphism on other traits, we examined the effects of additional stresses, including the addition of the drugs caffeine and rapamycin and relatively high concentrations of copper, salt, and ethanol to yeast-rich medium (YPD, 2% glucose). In four of the six conditions (the rich medium control, copper, caffeine, and rapamycin), a single-locus scan revealed a highly significant QTL peak in the same region of chromosome XV that included *SER1* (Figure 2). The two remaining conditions, ethanol and salt, had modestly significant QTL peaks at this locus, but shared a stronger peak on chromosome IV (Figure 2 and Table S9). The ethanol condition also showed another significant peak on chromosome IX (Figure 2 and Table S9).

Three conditions that consist of YPD plus an additive (copper, caffeine, or rapamycin) had LOD profiles that were nearly indistinguishable from YPD, raising the possibility that the phenotypic differences between the progeny merely reflected the growth differences in YPD. We examined this possibility in two ways: first by comparing the magnitude and direction of effect of the sake and tecc alleles in YPD *vs.* the other conditions, and second by statistically testing whether there was an interaction between the peak XV QTL marker and each condition. We first defined two subpopulations based on the allele at the peak XV QTL marker. Because of a clear floor effect in the progeny strains on rapamycin and caffeine, particularly in the sake subpopulation, we first performed quantile normalization on the two subpopulations (*Materials and Methods*).

After normalizing the rapamycin and caffeine phenotype values, we estimated the levels of broad-sense heritability and found them to be very high in all four of these YPD-based traits (YPD = 0.95, copper = 0.97, rapamycin = 0.99, and caffeine = 0.99). The proportion of the genetic variance explained by the *SER1* locus varied considerably across the four conditions, but was substantial in each (YPD = 0.23, copper = 0.26, rapamycin = 0.51, and caffeine = 0.48). Therefore, in all four of these conditions, growth is a highly heritable trait with the phenotype of any strain substantially determined by the allele of *SER1* that it carries.

The copper condition is a clear example of a case in which the added stress had relatively little impact on the growth of the progeny beyond that of YPD alone. The growth of the progeny on YPD *vs.* copper (Figure S2) shows a strong correlation (Pearson’s correlation coefficient = 0.945). Furthermore, the difference in mean growth between the subpopulations on YPD was 9 mm^2^, which is very similar to the 10.5 mm^2^ on copper (Figure S3). In contrast, the difference in mean growth between the subpopulations was 18.7 mm^2^ on rapamycin and 12.4 mm^2^ on caffeine. To test for an interaction between *SER1* and the growth medium in each of these three conditions, we carried out a two-factor ANOVA with an interaction term. Significant interactions were identified for rapamycin (*P* = 5.3 × 10^−19^) and caffeine (*P* = 3.7 × 10^−5^), but not for copper (*P* = 0.069). Taken together, our results are consistent with the hypothesis that factors regulated by the target of rapamycin (TOR) pathway contribute to the relatively robust growth of the auxotrophic sake strain in the presence of abundant nutrients.

**Figure 3:**
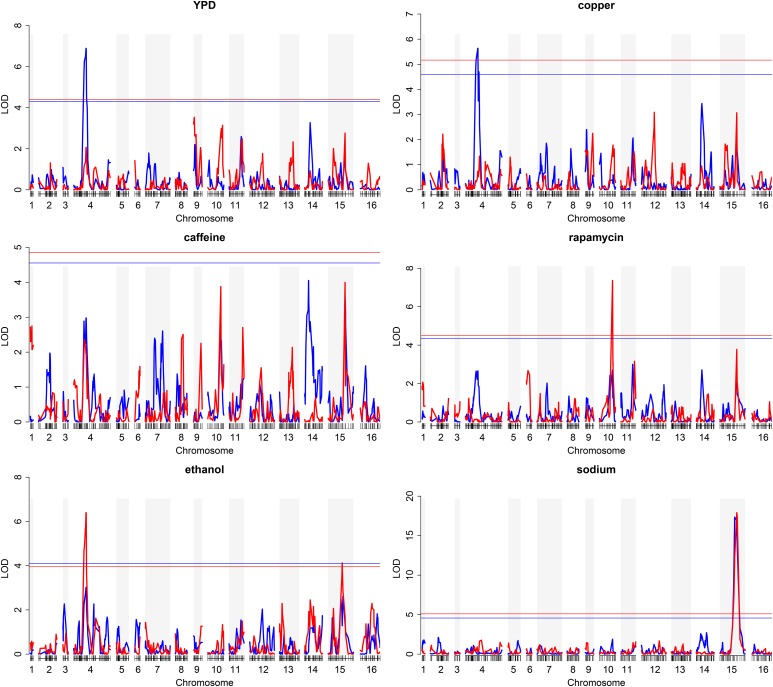
QTL mapping after fixing the major effect locus reveals minor-effect QTL. To identify loci that might be obscured by the major-effect locus in each condition, the cross population was split based on the genotype at the peak marker (denoted by * in Figure 2) and mapping was applied to each subset separately (blue = sake; red = tecc). Significance thresholds (red and blue horizontal lines) at a nominal α = 0.01 with Bonferroni correction (*n* = 6 conditions × 2 subsets = 12) were calculated separately for each subset by permutation (*n* = 1000).

Although *SER1* accounted for a substantial proportion of the genetic variance in all four of the YPD-based growth conditions, the fact that substantial residual genetic variance remained after accounting for *SER1* (YPD = 36.0, copper = 47.2, rapamycin = 81.9, and caffeine = 38.4; variance units are phenotype units squared, *i.e.*, mm^4^) suggested the existence of additional QTL. Because the large-effect size of *SER1* could mask detection of weaker secondary QTL, we split the populations based on the major-effect QTL and performed QTL analysis on each of the subpopulations (*Materials and Methods*). Similarly, for the ethanol and sodium conditions, the population was split on the major chromosome IX and IV QTL, respectively. This analysis identified several minor-effect QTL in the split populations that, with the exception of the rapamycin condition, were in the same locations as primary peaks identified in other conditions (Figure 3). For example, the secondary QTL peak linked to growth on YPD was located at the same position on chromosome IV as the major-effect peak observed on salt. Conversely, on salt, the secondary QTL peak linked to growth was located in the same region of chromosome XV as the major-effect peak observed in YPD. Thus, the only additional QTL uncovered by this stepwise analysis was a peak on chromosome X centered over *TOR1*, a core component of the TOR complex 1 (TORC1) (Heitman *et al.* 1991; Loewith *et al.* 2002), identified in the rapamycin condition (Figure 3). While we did not characterize the gene(s) underlying this QTL further, we note that the sake allele of *TOR1* harbors several nonsynonymous amino acid substitutions at highly conserved residues in the HEAT repeats or FAT regions of the protein (Table S10).

### SER1 is the causative gene underlying growth defects in several rich media conditions

**Figure 4:**
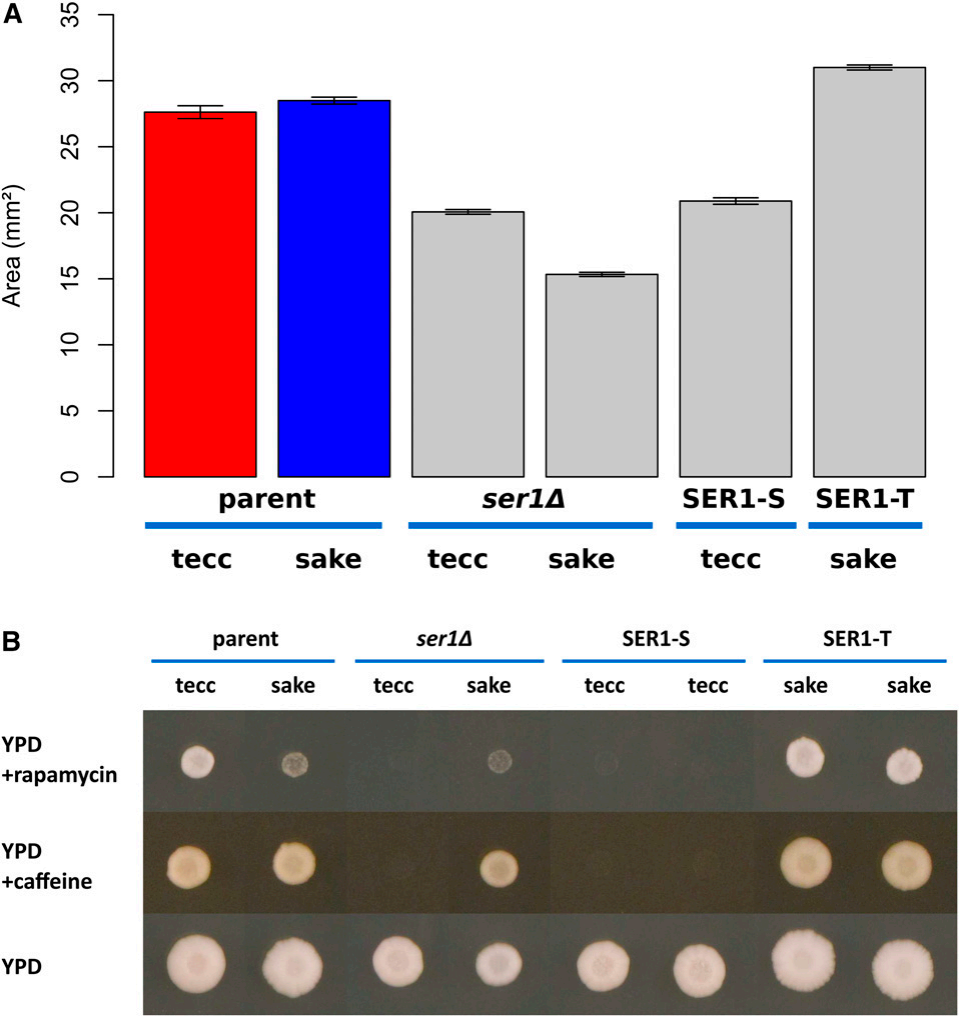
Allele swaps of *SER1*. (A) A comparison of patch area on YPD after 3 d of growth. Error bars are SE. (B) Representative example images of growth of these same strains on YPD + 0.1 µg/ml rapamycin, YPD +10 mM caffeine, and YPD. Strains include the parent strains of the cross (tecc and sake), both strains with the *SER1* open reading frame deleted (*ser1*Δ), and two independent isolates of each strain harboring allele replacements of *SER1*. Strains were photographed after 3 d of growth at 30°. Plate images were cropped without resizing or additional image manipulation to create the composite image presented. In both (A) and (B), each strain was assayed at least four times across two replicate plates for each condition.

To test whether *SER1* was also the causative gene underlying the QTL peaks in rich media conditions, we assayed the growth of the *ser1*Δ and allele-replacement strains in YPD, caffeine, and rapamycin (Figure 4). Our results demonstrated that the robust growth of the tecc genetic background was dependent on the presence of a functional *SER1* allele, with reduced or even no growth observed in a *ser1*Δ or a *SER1-S* allele replacement on YPD alone (Figure 4A) or in the presence of rapamycin or caffeine (Figure 4B). These results support the hypothesis that *SER1-S* is a loss-of-function allele and that the tecc genetic background provides a reliable readout of Ser1 activity. Results in the sake background were more complicated. In the presence of rapamycin, a comparable amount of growth was seen in the sake parental strain and a *ser1*Δ, but robust growth was conferred by the *SER1-T* allele replacement (Figure 4B). Thus, *SER1* is the causative gene underlying the growth defect in rapamycin. However, in both YPD alone and in the presence of caffeine, the sake parental strain exhibits stronger growth than would be predicted from the growth of progeny strains harboring the sake allele at that locus (Figure 2 and Figure 4). One possible explanation for this transgressive segregation pattern is the presence of compensatory adaptation(s) to Ser1 deficiency in the sake strain. However, the absence of modifier QTL peaks in the YPD or caffeine conditions (Figure 2 and Figure 3) suggests that this trait may be highly complex, unlinked to the Mendelian segregation patterns of the nuclear chromosomes, or involve one or more nongenetic factors.

### Natural variation in the ENA6 promoter improves growth in salt

Robust growth on salt was associated with the inheritance of a genomic region on chromosome IV from the sake parent (Figure 2). Within the support interval of this peak, we identified a promising candidate, the *ENA* locus, which has been previously linked to sodium and lithium growth defects (Kim and Fay 2007; Warringer *et al.* 2011; Wilkening *et al.* 2014; Hou *et al.* 2016). Different strain backgrounds harbor one or more genes (*ENA1*, *ENA2*, *ENA5*, and *ENA6*) at this locus that encode a yeast P-type ATPase sodium and lithium exporter(s) (Rudolph *et al.* 1989; Haro *et al.* 1991; Wieland *et al.* 1995). The parental strains of our cross each bear a single *ENA* gene that is homologous to the ancestral *S. cerevisiae ENA6* (Daran-Lapujade *et al.* 2009; Strope *et al.* 2015), although the tecc allele of *ENA6* shares homology with the hybrid *ENA1* allele over the last ∼465 bp of the gene (Table S11). Hereafter, we refer to the sake and tecc *ENA* alleles as *ENA6-S* and *ENA6-T*, respectively.

To test whether *ENA6* was in fact the causative gene underlying the QTL associated with growth in the salt stress condition, we constructed a series of deletion and allele-replacement strains and assayed their growth in the presence of high salt. Consistent with previous studies (Haro *et al.* 1991; Daran-Lapujade *et al.* 2009; Warringer *et al.* 2011), deletion of the *ENA6* open reading frame from the tecc genetic background significantly reduced growth on salt (Figure 6B). Surprisingly, despite the presence of numerous nonsynonymous coding polymorphisms between the two alleles (Table S11), replacement of the *ENA6-T* open reading frame with the sake coding sequence (*ENA6-pT-S*) did not restore growth on salt to the levels seen in the sake strain (Figure 6B).

**Figure 5:**
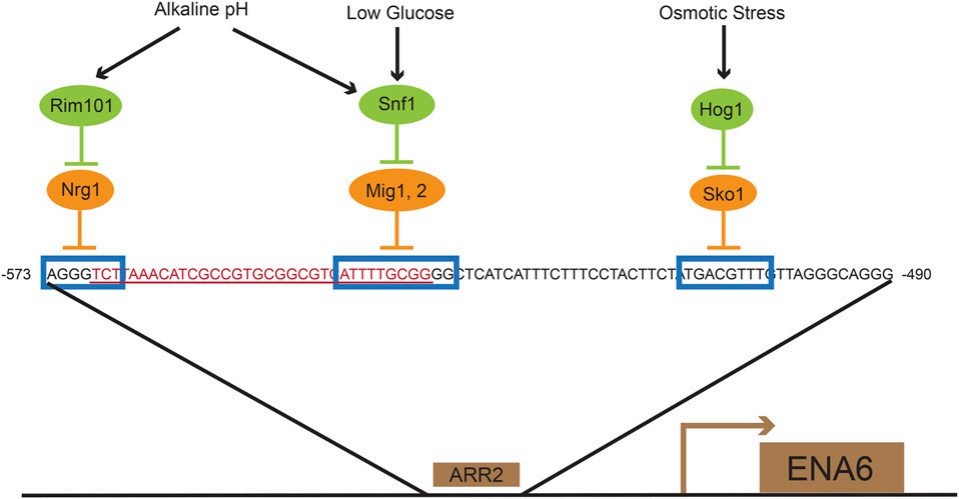
The ARR2 sequence within the *ENA6* promoter. Boxes indicate binding sites for repressor proteins Nrg1, Mig1, Mig2, and Sko1. Under standard growth conditions, *ENA6* is constitutively repressed at multiple locations within this region; however, a 33-bp deletion in the sake background (underlined in red) disrupts Nrg1 and Mig1, Mig2 binding sites. Figure modified from Serrano *et al.* (2002) and Ruiz and Arino (2007).

Given the previously observed relationship between salt tolerance and *ENA* expression levels (Daran-Lapujade *et al.* 2009; Warringer *et al.* 2011), we examined the sequence of the *ENA6* promoter regions. *ENA1* is constitutively repressed by numerous pathways including the HOG, glucose, calcineurin, and Rim101 pathways (Ruiz and Arino 2007). The zinc finger binding proteins Mig1 and Mig2 play a primary role in the glucose repression pathway (Lutfiyya *et al.* 1998), and deletion of *MIG1* and *MIG2* have additive effects on *ENA1* expression levels (Proft and Serrano 1999). *ENA1* is also repressed by Nrg1, a protein regulated by both the glucose repression pathway (Vyas *et al.* 2001) and the Rim101 pathway in response to high salt and high pH (Platara *et al.* 2006). Similar to the effect of deleting *MIG1*, deletion of *NRG1* is associated with the increased transcription of *ENA1* even in nonstress conditions (Lamb and Mitchell 2003). Interestingly, *ENA6-S* contains a 33-bp deletion (Figure 5) in a previously defined URS of the alkaline responsive region (ARR2) (Serrano *et al.* 2002) that deletes the Mig1 and Nrg1 binding sites (Proft and Serrano 1999; Serrano *et al.* 2002; Platara *et al.* 2006; Ruiz and Arino 2007).

**Figure 6:**
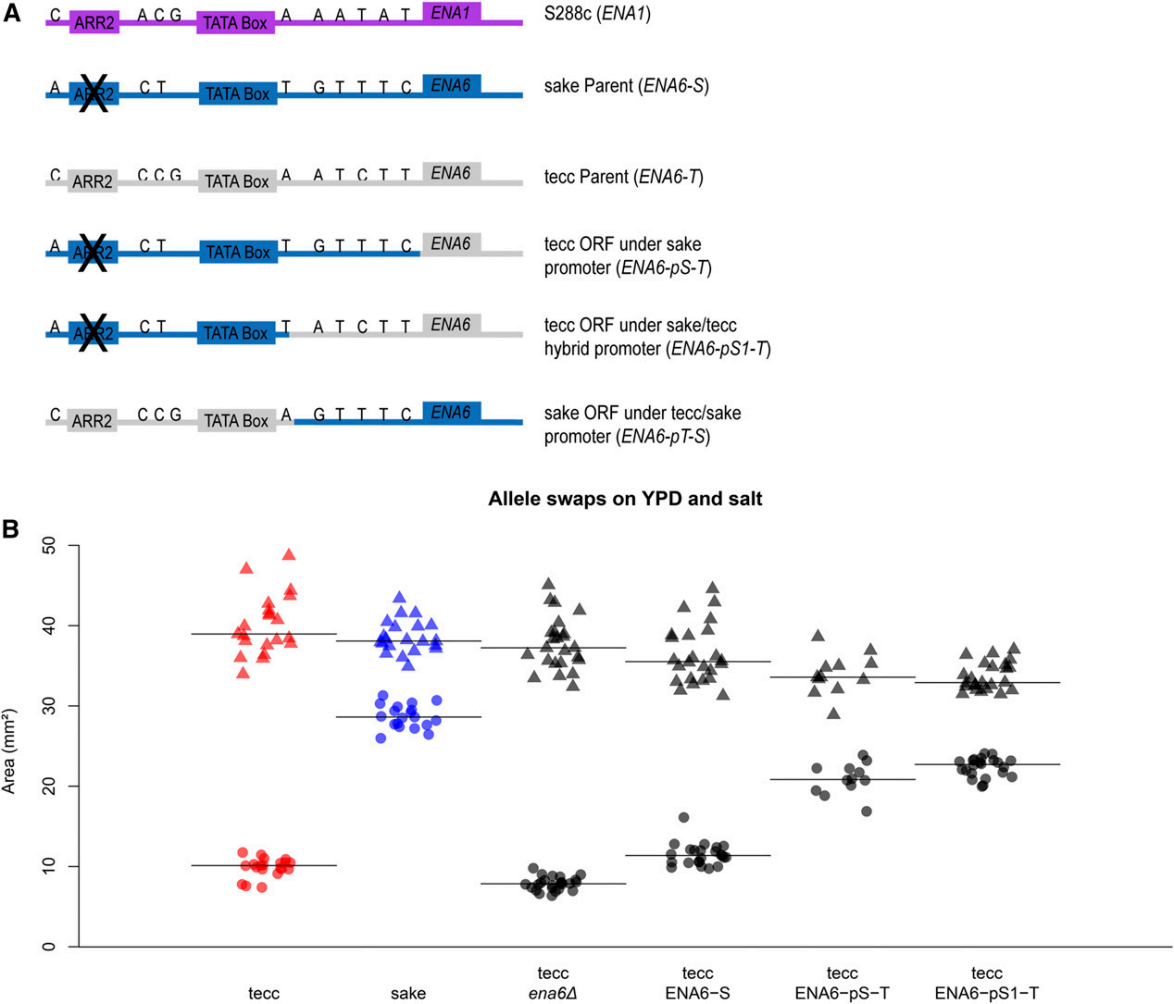
Polymorphisms in the *ENA6* promoter confer differential sensitivity to high salt. (A) A depiction of the promoter region of the sake (blue) and tecc (gray) *ENA6* alleles tested in this study compared to the reference strain (purple). The sake genetic background has a 33-bp deletion in the ARR2 URS which deletes binding sites for the Mig1, Mig2, and Nrg1 repressors. Representative images of additional tecc and sake background strains are shown in Figure S4. (B) The parental strains, a tecc *ena6*Δ, and three different allele substitution strains in the tecc background were grown on YPD (▴) and YPD + 0.5 M salt (●). The median of each group is indicated with a line. Each condition included 12 technical replicates (four replicates per plate and three plates per condition). In addition, all modified strains (black) had two biological replicates except tecc ENA6-pS-T, which had one biological replicate.

To test the effect of the promoter polymorphisms, we constructed a series of strains harboring the tecc *ENA6* open reading frame under the transcriptional control of the sake *ENA6* promoter and assayed their growth on high salt. Interestingly, alleles that replaced the tecc *ENA6* promoter with the sake promoter sequence (*ENA6-pS-T* and *ENA6-pS1-T*) rescued growth on high concentrations of salt (Figure 6B and Figure S4), demonstrating that the *ENA6* locus is the causative QTL involved in salt sensitivity in the tecc background and that this phenotype is strongly influenced by variation in the *ENA6* promoter region. Because extended homology between the allele swap cassette and the genomic sequence permitted recombination at different points across the promoter and produced some sake/tecc promoter hybrids (*Materials and Methods*), we were able to compare the effects of alleles that replaced only a portion of the promoter against the effects of replacing the entire 610-bp region upstream of the start codon (Figure 6 and Table S4). Strains harboring sake *ENA6* SNPs from −610 to −234 bp exhibited only slight differences in growth on high salt relative to strains with the entire 610-bp sake *ENA6* promoter region. These results further narrowed the causal regulatory region of *ENA6* to the large 33-bp deletion that removed the Mig1 and Nrg1 binding sites and seven other SNPs, including a 4-bp difference in the TA repeat length of the TATA box.

### Small, but condition-specific effects of aneuploidy

RAD-seq genotyping of the progeny (*Materials and Methods*) uncovered an extra copy of chromosome I from the sake parental strain that segregated at 2:2 through the cross, but with an overrepresentation of chromosome I heterozygosity in the aneuploid progeny (Table S6 and Table S12). To assess whether the chromosome I disomy affected growth, we compared growth of the euploid and aneuploid strains in each condition. Because some of the conditions had nonnormal phenotype distributions, we used a nonparametric statistical test (Mann–Whitney *U*-test). We found no significant difference between the two populations on YPD, copper, salt, rapamycin, and SD (α = 0.01 with Bonferroni correction *n* = 7). Statistically significant differences were found in ethanol (*P* = 0.0012) and caffeine (*P* = 0.0004). The mean difference in growth between chromosome I monosomes and disomes were 1.05 mm^2^ on ethanol and 2.54 mm^2^ on caffeine. Based on these results, we conclude that the chromosome I disomy had a small, but condition-specific effect on the progeny of this cross.

## Discussion

Our study uncovered a rare polymorphism in the yeast ortholog of the second enzyme in the serine biosynthesis pathway, phosphoserine aminotransferase (*SER1*). Serine has roles in numerous metabolic pathways linked to cellular growth and proliferation. It is a main donor of one-carbon units fueling the folate cycle, methionine cycle, and transsulfuration pathway; and the serine biosynthesis pathway is interconnected with glucose metabolism (Appling 1991; Christensen and MacKenzie 2006; Kalhan and Hanson 2012; Lee *et al.* 2013; Ducker and Rabinowitz 2017). The genes encoding enzymes involved in serine biosynthesis, such as 3-phosphoserine aminotransferase, are highly conserved from yeast (*SER1*) to human (*PSAT1*). This includes the Ser1 residue G78 (G77 of Psat1) responsible for the loss-of-function mutation studied here. Overexpression of serine pathway enzymes in neoplastic cells is thought to drive oncogenesis in a number of human cancers (Possemato *et al.* 2011; Locasale 2013; DeNicola *et al.* 2016). Loss-of-function mutations in these enzymes are linked to a rare class of inborn errors of metabolism known as serine deficiency disorders in which the spectrum of phenotypes is directly associated with the bioavailability of serine (Tabatabaie *et al.* 2010; El-Hattab *et al.* 2016). Given that *S. cerevisiae* preferentially imports amino acids when nutrients are available (Campbell *et al.* 2015), it is perhaps surprising that a serine auxotrophy would have such a large effect in rich media conditions. However, our results with this naturally occurring auxotrophy’s effect on growth are consistent with the widespread effects of engineered gene deletions on transcriptional profiles of cells grown in rich conditions (Ronald and Akey 2007; Alam *et al.* 2016), and thus underscore the need to carefully consider the use of auxotrophic markers in genetic analysis.

The sake loss-of-function allele *SER1-S* also confers a growth defect in the parents and progeny of this cross when grown in the presence of rapamycin, the immunosuppressive and antiproliferation drug which blocks cell cycle progression by inhibiting TORC1 (Heitman *et al.* 1991). Conserved across eukaryotes, TORC1 is the hub of a complex signaling network that integrates nutrient availability with cell growth and proliferation (Loewith *et al.* 2002; Goberdhan *et al.* 2016). Our results are consistent with the strong growth defects of the reference strain (Winston *et al.* 1995) observed in high throughput screens of the MAT**a** haploid deletion collection (Parsons *et al.* 2004) and homozygous diploid deletion collection (Hillenmeyer *et al.* 2008). One possible explanation for the strong interaction between the *SER1-S* allele and rapamycin is the significant decrease in amino acid transport by yeast in response to rapamycin (Schmidt *et al.* 1998; Beck *et al.* 1999; Gonzalez and Hall 2017).

Our results also identified *ENA6* as the causative gene underlying growth differences in the presence of high salt. The *ENA* locus is highly polymorphic in *S. cerevisiae*. Because it is the site of a recent introgression of sequence from *S. paradoxus*, many strain backgrounds, including the S288c-derived reference strain background (Winston *et al.* 1995; Goffeau *et al.* 1996), harbor multiple copies of hybrid *S. cerevisiae ENA6* and the *S. paradoxus ENA* genes (Wieland *et al.* 1995; Doniger *et al.* 2008; Strope *et al.* 2015). Strains with multiple *ENA* genes at this locus (*e.g.*, *ENA1*, *ENA2*, and *ENA5*) exhibit higher salt tolerance than strains with a single copy of *ENA6*, although overexpression of *ENA6* can rescue growth on high concentrations of salt (Daran-Lapujade *et al.* 2009; Warringer *et al.* 2011; Wilkening *et al.* 2014). Here, we identify a naturally occurring polymorphism in the *ENA6* promoter, in which growth in the presence of high salt is increased in large part due to the deletion of a transcriptional repressor element. Thus, natural isolates of *S. cerevisiae* have evolved several different mechanisms of increasing expression of the ENA transporters under specific environmental conditions.

## Supplementary Material

Supplemental material is available online at www.g3journal.org/lookup/suppl/doi:10.1534/g3.117.300392/-/DC1.
