# Supplementary figures and images for: Natural Variation in SER1 and ENA6 Underlie Condition-Specific Growth Defects in Saccharomyces cerevisiae
Source: G3 (Bethesda). 2017 Nov 14;8(1):239–51. doi: 10.1534/g3.117.300392 (PMC5765352; doi:10.1534/g3.117.300392)

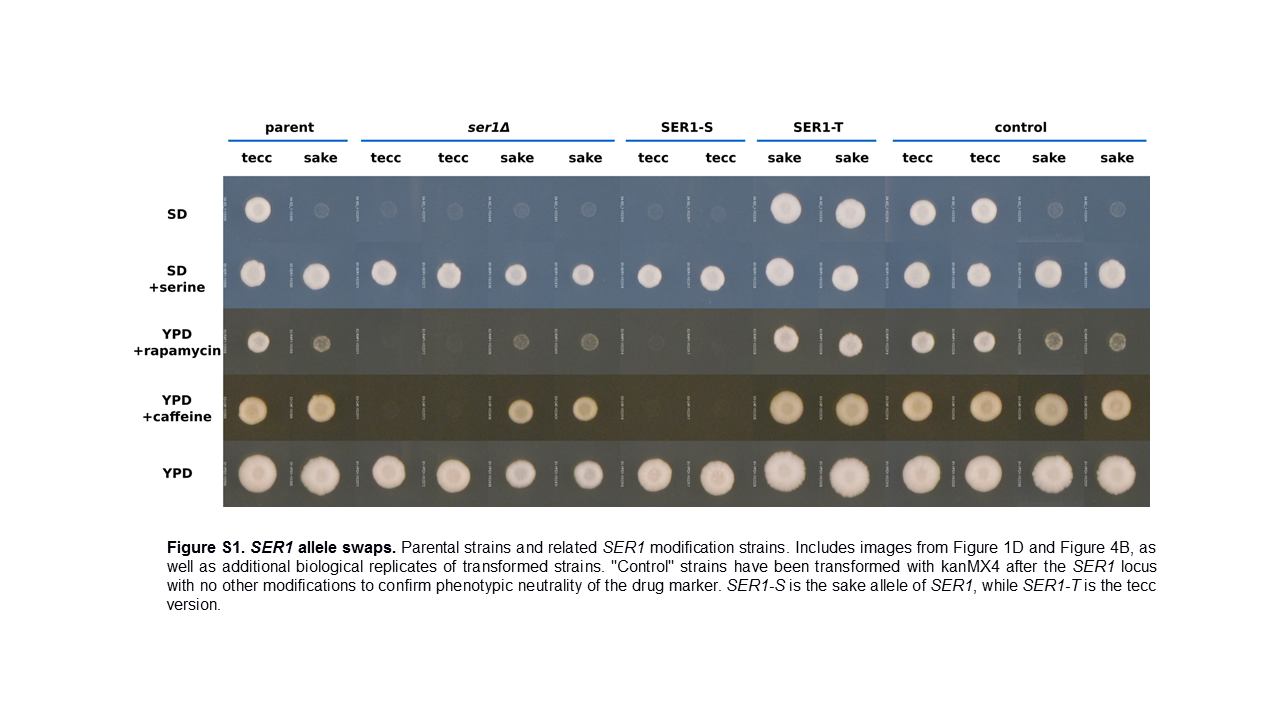

Supplement: Supplementary file 1 [file 239FigureS1.tif]

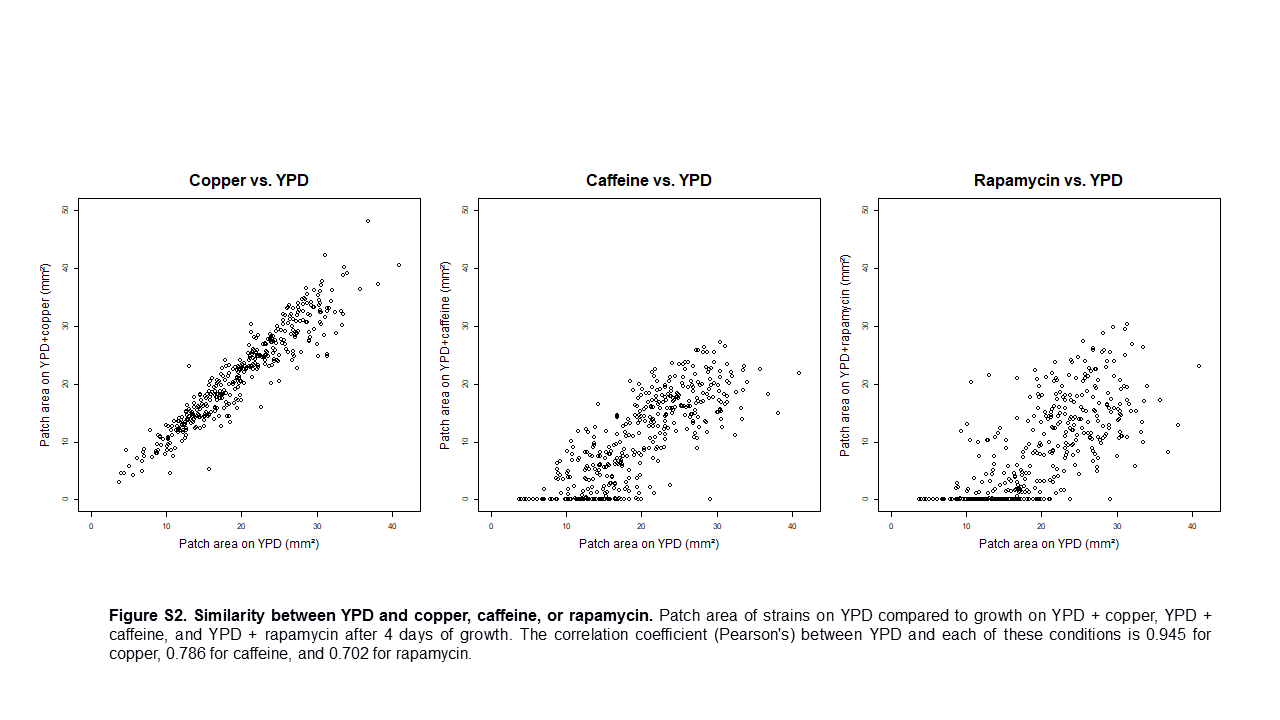

Supplement: Supplementary file 2 [file 239FigureS2.tif]

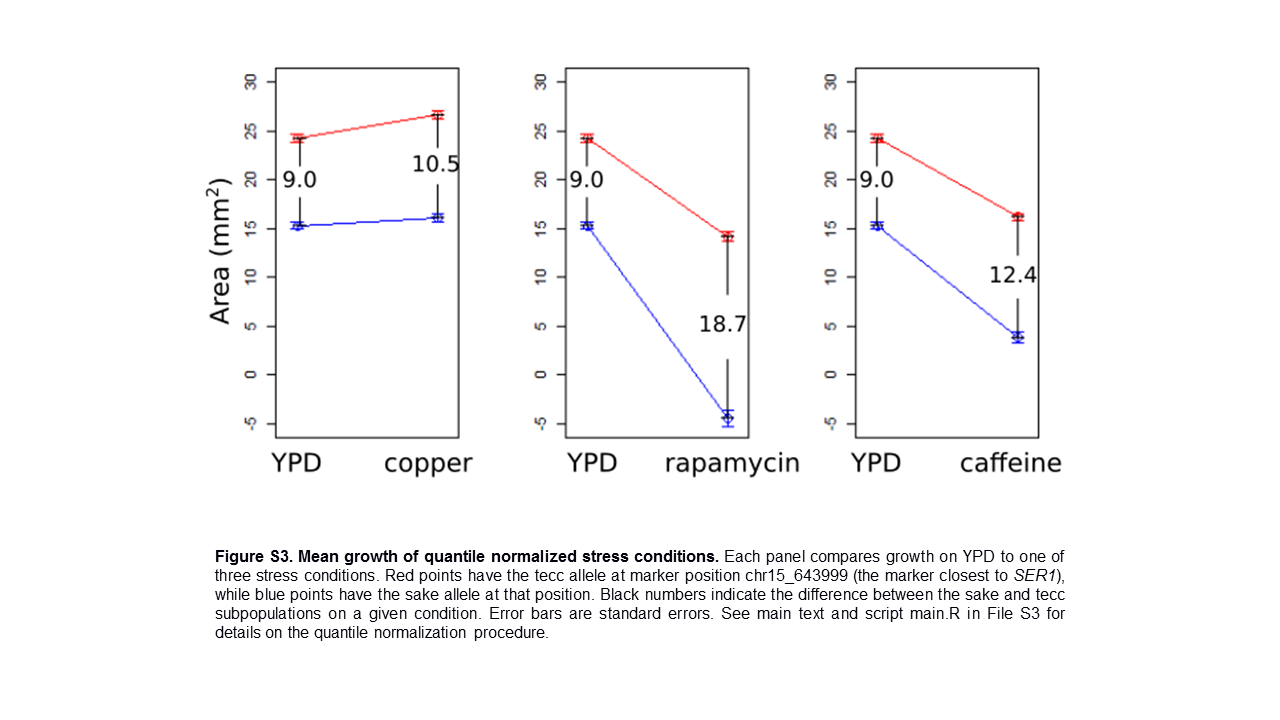

Supplement: Supplementary file 3 [file 239FigureS3.tif]

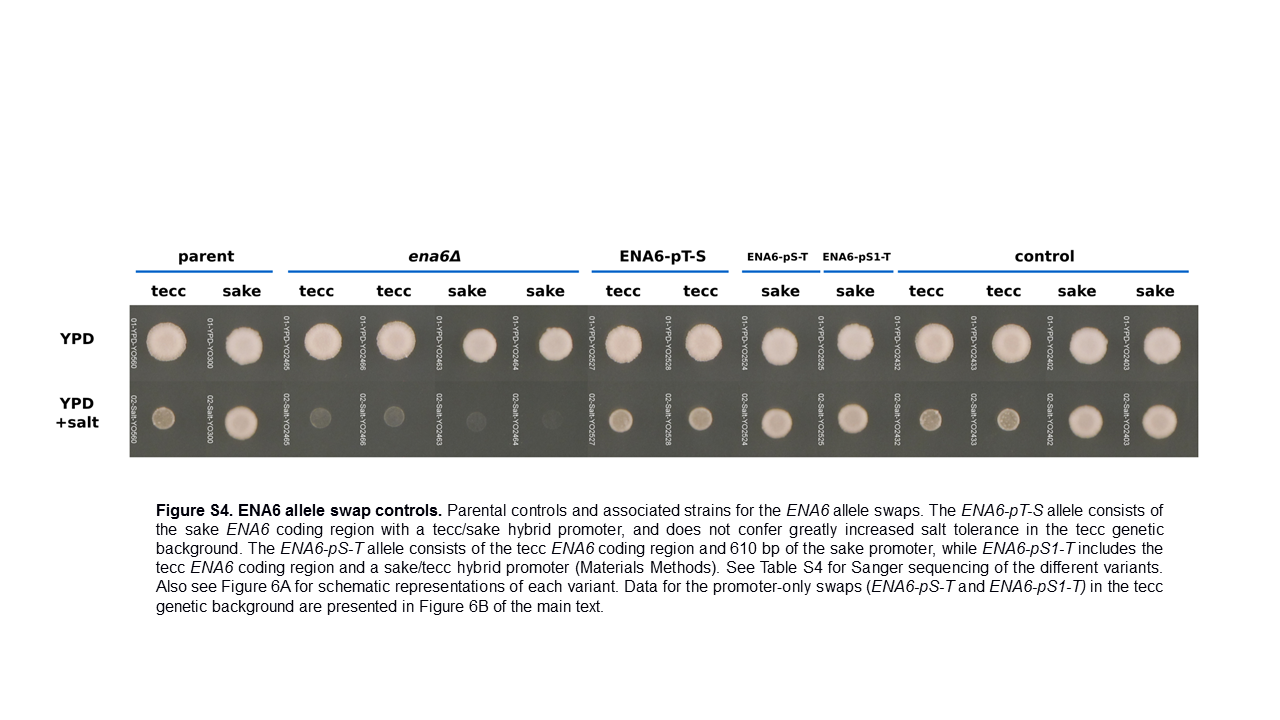

Supplement: Supplementary file 4 [file 239FigureS4.tif]
