# Supplementary material for: Natural Variation in SER1 and ENA6 Underlie Condition-Specific Growth Defects in Saccharomyces cerevisiae
Source: G3 (Bethesda). 2017 Nov 14;8(1):239–51. doi: 10.1534/g3.117.300392 (PMC5765352; doi:10.1534/g3.117.300392)
Supplement: Supplementary file 17 [file 239FileS1.docx]

**SUPPLEMENTAL FILES**

File S2. ImageJ script to extract patch areas from images.

File S3. Archive with R script to build R/qtl object from included data files.

**SUPPLEMENTAL FIGURES**

Figure S1. Images of all SER1 swaps

Figure S2. Phenotype scores of YPD vs. copper, YPD vs. caffeine and YPD vs. rapamycin.

Figure S3. Mean growth of quantile normalized subsets of progeny on YPD, copper, rapamycin, and caffeine.

Figure S4. Images of additional ENA6 allele swaps.

**SUPPLEMENTAL TABLES**

Table S1. Primers used in this study

Table S2. Alignment of BY4741, sake and tecc *SER1* alleles.

Table S3. HiFi Assembly fragments and primers

Table S4. Sanger sequence of the *ENA6* alleles

Table S5. SNP table

Table S6. Estimated ploidy of each chromosome for each strain

Table S7. Progeny strain genotypes and phenotypes.

Table S8. Quantile normalized rapamycin/caffeine phenotypes

Table S9. LOD scores and marker locations.

Table S10. SNPs within coding region of *TOR1*

Table S11. Amino acid alignment of sake and tecc *ENA6* to reference alleles

Table S12. HomA/HomB/Het calls for chr01 disomes. Includes all strains (including euploids) so that this file can be easily aligned with Table S7.
